# Supplementary figures and images for: Curcumin Attenuates the Pathogenicity of Entamoeba histolytica by Regulating the Expression of Virulence Factors in an Ex-Vivo Model Infection
Source: Pathogens. 2019 Aug 15;8(3):127. doi: 10.3390/pathogens8030127 (PMC6789811; doi:10.3390/pathogens8030127)

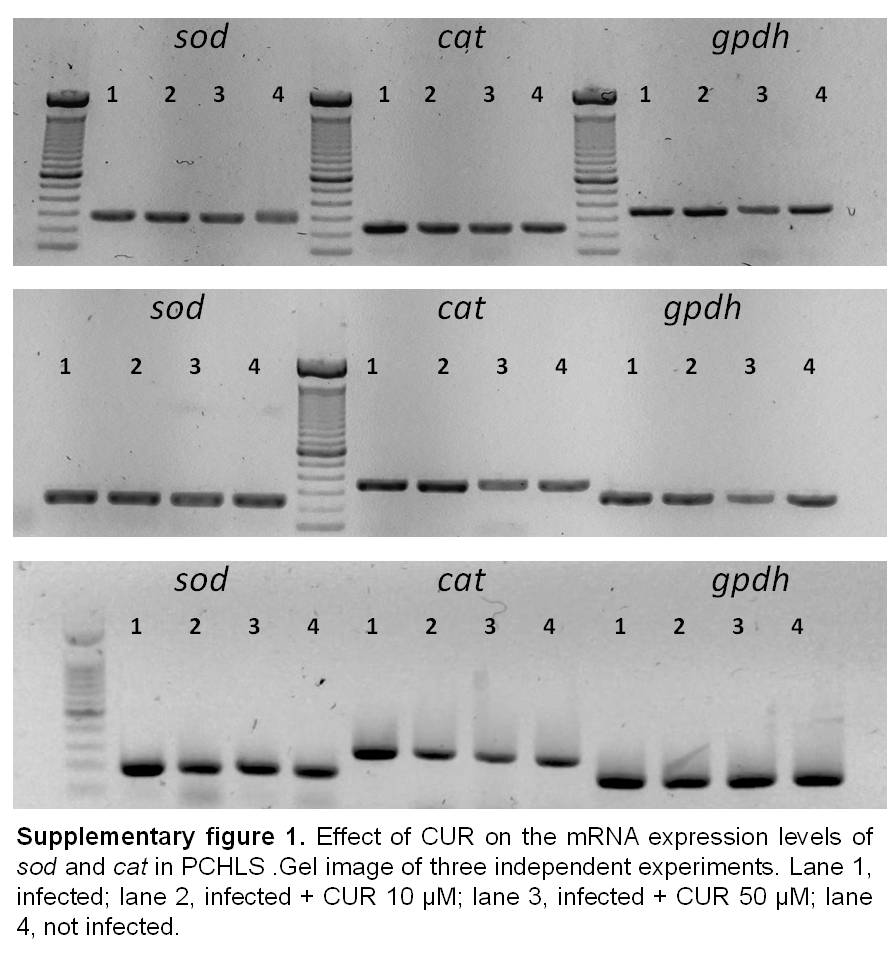

Supplement: Supplementary file 1 [file pathogens-08-00127-s001.zip › Supplementary figure 1.jpg]

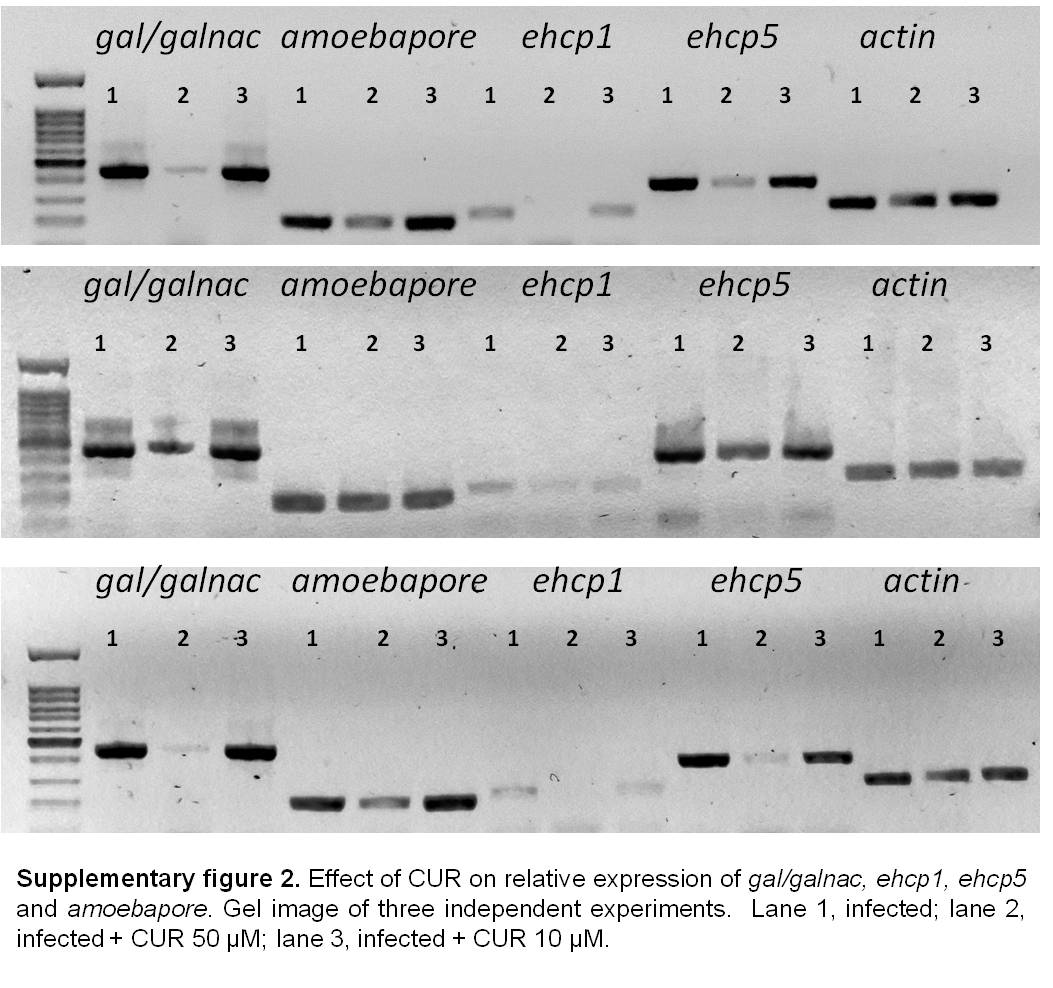

Supplement: Supplementary file 1 [file pathogens-08-00127-s001.zip › Supplementary figure 2.jpg]
